# A case report of dyspnea that revealed a congenital left cardiac diverticulum of discovery in adulthood with a fatal course

**DOI:** 10.1016/j.ijscr.2025.111223

**Published:** 2025-03-27

**Authors:** Fethi Jebali, Asma Ladib, Mohamed Ali Chaouch, Saida Hidouri, Hafedh Daly, Rachid Ben Soussia

**Affiliations:** a Department of Anesthesia, Monastir Maternity University Hospital, Monastir, Tunisia; b Department of Visceral and Digestive Surgery, Monastir University Hospital, Monastir, Tunisia; c Department of Pediatric Surgery, Hospital of Zaghouan, Tunisia; d Department of Vascular Surgery, Monastir University Hospital, Monastir University, Tunisia; e Department of Anatomy, Monastir Faculty of Medicine, Monastir University, Tunisia

**Keywords:** Cardiac diverticulum, Aneurysm, Dyspnea, Congenital heart disease, Case report

## Abstract

**Introduction and importance:**

Congenital cardiac diverticula are rare malformations involving the myocardium, the endocardium, and occasionally the pericardium. They have variable presentations and are often incidental findings.

**Case presentation:**

A 61-year-old man with no significant history presented worsening exertional dyspnea. Examination revealed obesity but was otherwise unremarkable. Initial tests were normal, but chest radiography showed cardiomegaly and ECG indicated diffuse microvoltage. Echocardiography revealed a large left ventricular diverticulum (7 × 8 cm) with a wide neck (4 cm) and severe dysfunction (EF 30 %). Coronary angiography confirmed normal coronary arteries. Despite intensive care, his condition deteriorated and required mechanical ventilation and vasoactive support. He underwent emergency surgery but succumbed intraoperatively.

**Clinical discussion:**

Although rare, congenital cardiac diverticula can cause severe complications, including heart failure and sudden death. Echocardiography is the key to diagnosis, with CT or MRI providing further characterization. Surgery is the preferred treatment for symptomatic cases, while asymptomatic patients require close follow-up.

**Conclusion:**

This case highlights the need for early diagnosis and timely intervention in congenital cardiac diverticula to prevent fatal outcomes.

## Highlights

• Congenital left cardiac diverticulum is a rare anomaly often discovered incidentally, but in this case, it presented with severe dyspnea and a rapidly deteriorating clinical course in adulthood.
• Echocardiography, coronary angiography, and ventriculography played a crucial role in diagnosing the large left ventricular diverticulum, highlighting the importance of multimodal imaging.
• Despite intensive medical and surgical intervention, the fatal outcome emphasizes the need for early diagnosis, timely surgical management, and vigilant follow-up in patients with congenital cardiac diverticula.

## 1 Introduction

Congenital cardiac diverticula are a rare malformation formed by saccular evagination of the ventricular wall of the heart, including the myocardium, the endocardium, and occasionally the pericardium [1]. They can be of very different sizes and locations and are also the occasion for very different clinical manifestations [2,3]. Diverticula can be muscular or fibrous and affect any heart chamber, including the left ventricle, and are most often associated with other malformations. This case report, reported according to SCARE guidelines [4] aims to describe a rare presentation of congenital left cardiac diverticulum discovered in adulthood, which led to a fatal outcome. The report highlights clinical manifestations, diagnostic findings, and challenges in managing this condition, emphasizing the importance of early recognition and appropriate intervention to prevent adverse outcomes.

## 2 Case presentation

**Fig. 1:**
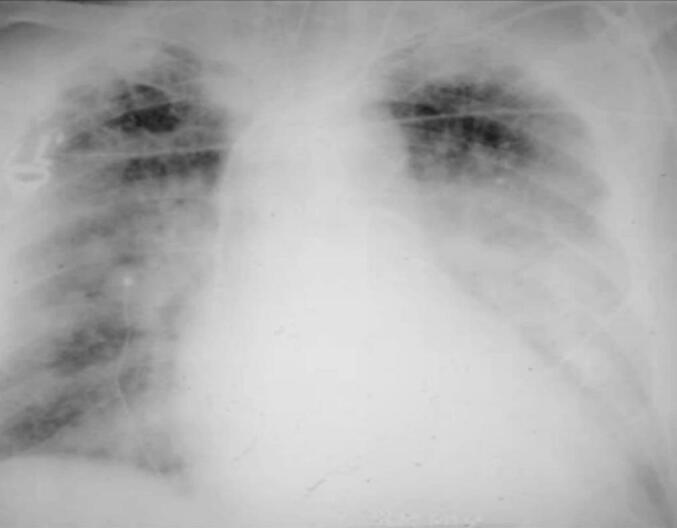
A chest X-ray showing an enormous cardiomegaly.

**Fig. 2:**
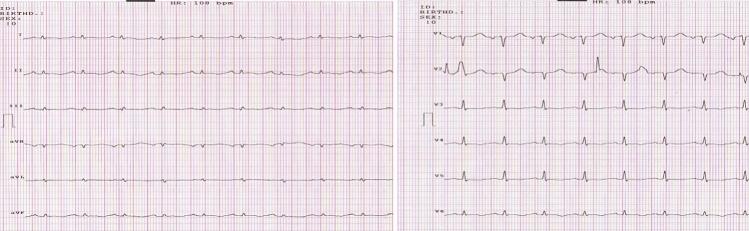
The electrocardiogram.

**Fig. 3:**
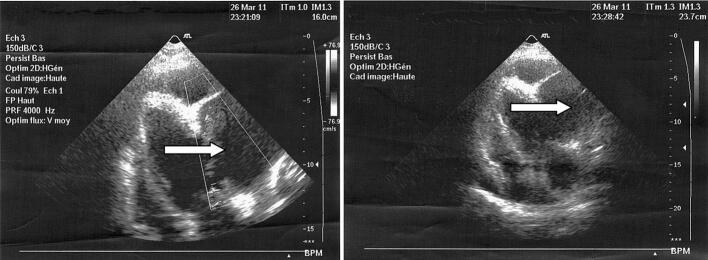
Cardiac echography showing the four cardiac cavities (white arrow).

A 61-year-old man, with no significant medical history and no family history of congenital heart disease, presented to the emergency department with worsening dyspnea, particularly on exertion. On physical examination, findings were unremarkable except for android obesity. His blood pressure was 130/80 mmHg, with a regular pulse. Cardiac auscultation revealed no murmurs or additional sounds, while pulmonary auscultation detected crackles at the lung bases. Laboratory tests, including arterial blood gas analysis, were within normal limits. A chest radiograph revealed significant cardiomegaly (Fig. 1). Electrocardiography (ECG) showed diffuse microvoltage, regular sinus rhythm, and no conduction, repolarization, or rhythm abnormalities (Fig. 2). Transthoracic echocardiography confirmed the diagnosis, identifying a large left ventricular diverticulum measuring 7 × 8 cm, with a wide neck of 4 cm. The right cardiac chambers were not dilated, there were no signs of pulmonary arterial hypertension (PAH), but left ventricular function was severely impaired, with an ejection fraction (EF) of 30 % (Fig. 3). Subsequent coronary angiography demonstrated normal coronary arteries. Left ventriculography confirmed a large cavity connected to the left ventricle. The patient's condition rapidly deteriorated, with worsening dyspnea, hypoxia on arterial blood gas analysis, and bilateral alveolar syndrome on chest radiographs. His predominantly left-sided heart failure worsened, necessitating inotropic support. BNP levels showed a marked increase. Despite intensive medical management, his clinical condition deteriorated further, requiring intubation, mechanical ventilation, and vasoactive drugs. The patient was referred to for urgent surgery but suffered intraoperative cardiac arrest and could not be resuscitated.

## 3 Discussion

Congenital cardiac diverticulum, first described by O'Bryan in 1837 [1], is a rare cardiac malformation with an unclear embryological origin. It can have either a muscular or fibrous wall, with the former often being asymptomatic and discovered incidentally, while the latter is more prone to complications such as heart failure and life-threatening arrhythmias, including ventricular tachycardia and sudden cardiac death [2,3]. Two forms have been described: the intrathoracic form, which is rare and often diagnosed incidentally in adulthood through electrocardiographic abnormalities or chest radiographs, and the thoracoabdominal form, which is more frequent and associated with Cantrell syndrome, presenting as a pulsatile, expansive mass in the epigastric or subcutaneous region [2,5]. Diagnosis is primarily established via echocardiography, with CT and MRI providing detailed anatomical and functional characterization, differentiating diverticula from aneurysms or other pericardial abnormalities [3,6]. When faced with a cystic or bulging lesion adjacent to the left ventricle, the differential diagnosis should include several entities beyond the congenital ventricular diverticulum. Left ventricular aneurysms, whether congenital or acquired (e.g., post-infarction), often present similarly but typically have thinner walls and paradoxical motion, distinguishing them from contractile diverticula. Pseudoaneurysms, which lack myocardial tissue and are contained by pericardium or scar, pose a high rupture risk and require urgent surgical management. Pericardial cysts, although benign and extrinsic to the myocardium, can appear as similar radiologic findings and must be excluded through imaging modalities such as MRI or CT. Other rare differentials include ventricular clefts, which are narrow invaginations with systolic obliteration, and congenital cardiac tumors like fibromas or rhabdomyomas. Accurate differentiation is essential, as the management and prognosis vary significantly between these conditions. The management approach depends on symptom severity and associated cardiac abnormalities. Surgical resection is recommended for symptomatic patients, particularly those with hemodynamic compromise, arrhythmias, or severe ventricular dysfunction, though thoracoabdominal forms carry a more guarded prognosis. Conservative management with close follow-up is preferred for asymptomatic cases to avoid complications such as embolism, arrhythmias, heart failure, or rupture leading to sudden death. In recent years, radiofrequency ablation has been considered for small, arrhythmogenic diverticula. The unfavorable outcome, in this case, may be attributed to delayed diagnosis, surgical complexity due to the large size (7 × 8 cm) and wide neck (4 cm), and severe preoperative hemodynamic instability requiring vasoactive support, all of which are recognized risk factors for intraoperative mortality. Literature reports indicate higher survival rates (70–80 %) in elective surgical repairs, whereas emergency interventions in hemodynamically unstable patients carry a significantly higher mortality risk [4]. This case highlights the importance of early diagnosis and timely intervention to improve prognosis and prevent catastrophic complications.

## 4 Conclusions

This case highlights the need for early diagnosis and timely intervention in congenital cardiac diverticula to prevent fatal outcomes. In all cases, a medical surgical consultation is necessary. If surgical abstention is decided, regular ultrasound monitoring of the muscular diverticula is necessary given the possibility of its fibrous transformation.

## Consent

Written informed consent was obtained from the patient for the publication of this case report and accompanying images. A copy of the written consent is available for review by the Editor-in-Chief of this journal upon request.

## Ethical approval

Not applicable.

## Funding

This research did not receive specific grants from the public, commercial or not-for-profit sectors.

## Author contribution

All authors participated in the treatment of the patients, writing, and approving the manuscript.

## Guarantor

Mohamed Ali Chaouch.

## Research registration number

1. Name of the registry: N/A.
2. Unique identifying number or registration ID: N/A.
3. Hyperlink to your specific registration (must be publicly accessible and will be checked): N/A.

## Conflict of interest statement

No conflict of interest to disclose.
